# Diverse Bacterial Properties Influence Dispersal Along Fungal Networks

**DOI:** 10.3390/jof12060425

**Published:** 2026-06-11

**Authors:** Roberto Regalado, Mariana Santos Craveiro Silva, Euan Price, Nai-Wen Liang, Caroline M. Grunenwald, John-Demian Sauer, David J. Beebe, Nancy P. Keller

**Affiliations:** 1 Department of Plant Pathology, University of Wisconsin-Madison, Madison, WI 53706, USA; santoscravei@wisc.edu (M.S.C.S.); npkeller@wisc.edu (N.P.K.); 2 Department of Molecular Microbiology and Immunology, University of Missouri School of Medicine, University of Missouri, Columbia, MO 65211, USA; 3 Department of Biomedical Engineering, University of Wisconsin-Madison, Madison, WI 53706, USA; 4 Department of Biological Sciences, University of Missouri, Columbia, MO 65211, USA; cmgrunenwald@missouri.edu; 5 Department of Medical Microbiology and Immunology, University of Wisconsin-Madison, Madison, WI 53706, USA; sauer3@wisc.edu; 6 Department of Pathology and Laboratory Medicine, University of Wisconsin-Madison, Madison, WI 53706, USA

**Keywords:** fungal networks, *Aspergillus*, *Listeria*, *Staphylococcus*, flagella, phenol-soluble modulins, quorum-sensing

## Abstract

Bacterial–fungal interactions are prevalent in microbial communities, and fungi often facilitate bacterial dispersal along networks created by fungal hyphae. Using a microfluidic device, we examined how diverse bacterial species disperse in monoculture versus travel in coculture with *Aspergillus flavus*. Most of the bacteria traveled further when in coculture, although this was not absolute. Two bacteria showing significant dispersal rates only in coculture were the human pathogens *Listeria monocytogenes* and *Staphylococcus aureus.* Mechanistically, *L. monocytogenes* dispersal required flagella, with dispersal impaired in flagellar mutants but enhanced in *∆mogR* strains that upregulate flagellar expression. In contrast, the non-flagellar bacterium *S. aureus* exhibited a unique, wave-like dispersal pattern along the hyphae, a phenomenon that was abolished in *agr* quorum-sensing mutants deficient in phenol-soluble modulins (PSMs). In a triculture of *L. monocytogenes*, *S. aureus*, and *A. flavus*, *L. monocytogenes* limited *S. aureus* dispersal along the fungal hyphae; however, this inhibition was dependent on an intact *L. monocytogenes* quorum system. Our findings reveal that bacterial motility on fungal networks arises from diverse, species-specific mechanisms, including flagella, transcriptional regulation, potential quorum-sensing-mediated interactions, as well as other notable dispersal phenomena that warrant further investigation.

## 1. Introduction

Fungi and bacteria are ubiquitous across microbial communities, where they engage in diverse symbiotic interactions. Their interactions encompass all types of symbiosis [1]. A common arrangement of both microbes is found in mixed biofilm formation, such as *Bacillus subtilis* colonization of *Aspergillus niger* and *Agaricus bisporus* [2]. *Saccharomyces cerevisiae* and *Lactobacillus* spp., though unable to form cocultures by conventional methods, were shown to form structured biofilms in Daqu, a traditional Chinese fermentation starter [3]. Biofilms are found on biotic and abiotic surfaces in all environments, ranging from soils [4], abiotic structures [5], foods [6], and clinical settings [7]. Biofilms consist of heterogeneous mixtures where fungal mycelium can serve as a structural framework, with bacteria often adding a biological matrix of various polymeric substances. Individual fungal hyphae can act as dispersal aids, creating a fungal network that microorganisms can utilize [8]. This fungal network acts as a transport system, enabling migration of bacteria through various substrates. For example, *Pseudomonas aeruginosa* and other oxalotrophic bacteria have been shown to disperse via *Trichoderma* sp. mycelia to access an oxalate-rich medium [9]. The ability of *Serratia marcescens* D1 to invade and colonize fungal hyphae, particularly those of *Mucor irregularis*, suggests that endo-hyphal lifestyles may facilitate bacterial dispersal within fungal networks, leveraging the host’s mycelial structures as conduits for movement and expansion [10]. *B. subtilis* has been observed to utilize the fungal networks provided by *Aspergillus nidulans*. It employs the water layers encapsulating the hyphal surfaces to rapidly glide along these surfaces, achieving speeds of approximately 30 μm s^−1^ in the process [11]. Similarly, *Serratia proteamaculans*, along with other motile bacteria found in cheese rinds, utilize fungal networks by swimming within the liquid films that develop on fungal hyphae [12].

For some bacterial spp., flagella are known to assist bacterial dispersal on hyphal surfaces within the thin water films coating them. Flagellar motility in bacteria, though energetically costly, may be evolutionarily maintained in unsaturated soils due to the fitness advantage conferred by dispersal along fungal hyphae, as demonstrated by *Pseudomonas putida* KT2440 using the mycelial network of *Morchella crassipes* as a transport route [13]. In *Burkholderia terrae* strain BS001, flagella are critical for dispersal along fungal hyphae through soil, enabling swimming motility required for co-migration. In contrast, Type IV pili (T4P) play a minor role, supporting surface twitching motility but not being essential for fungal association. In soil microcosm experiments with *Lyophyllum* sp. and *Trichoderma asperellum*, flagellar mutants lost both motility and the ability to co-migrate with fungal partners, whereas T4P mutants showed only slightly reduced co-migration compared to the wild-type. These findings establish that fungal-assisted bacterial dispersal in *B. terrae* BS001 is flagella-dependent, with T4P contributing secondarily [14]. The movement of non-motile bacteria through fungal networks is largely unknown, though recent studies have begun to elucidate specific examples of this phenomenon [15].

In this study, to address the primary research question of how different bacterial species utilize a fungal hyphal network for dispersal and to determine if they are suggestive of specific molecular mechanisms governing these interactions, we probed dispersal strategies among diverse bacteria using *Aspergillus flavus*, a frequent soil inhabitant and seed pathogen known to form biofilms with bacteria [16,17,18]. We tested two specific hypotheses: first, that fungal hyphal networks facilitate bacterial dispersal, but the efficiency and mechanism are species-dependent, and second, that dispersal is dependent on bacterial traits, such as flagella, and can be modulated by inter-species quorum-sensing. Although no clear pattern emerged among these taxonomically distinct species—indicating that flagella, cell morphology, or Gram status do not universally predict dispersal patterns—we identified distinct traits of specific species that leverage movement along the fungal networks. Our findings revealed that, while flagellar mutants of *Ralstonia solanacearum* and *P. aeruginosa* showed little or no difference in dispersal compared to their wild-type counterparts, *Listeria monocytogenes* dispersal was flagella-dependent; flagellar mutants exhibited a notable decrease in dispersal, while a MogR mutant, a temperature-dependent transcriptional regulator of flagellin expression [19], showed increased motility. We further identified that quorum-sensing (QS) systems play a significant role in these interactions. In our model, an intact QS system was necessary for the dispersal of the non-motile species *S. aureus* along the fungal network. Furthermore, we observed that an intact QS system in *L. monocytogenes* could inhibit the dispersal of *S. aureus* in triculture with *A. flavus*, while a disrupted system could not, suggesting a complex inter-species competitive dynamic.

## 2. Materials and Methods

### 2.1. Fungal and Bacterial Cultures

The microbial species used in this study include a range of environmental and laboratory strains of fungi and bacteria. For fungal cocultures, *A. flavus* was used. Bacterial species included *S. aureus*, *L. monocytogenes*, *R. solanacearum*, *Gluconobacter* sp., *Bacillus velezensis, Burkholderia unamae*, *Micrococcus luteus*, and *P. aeruginosa*. All microbial isolates were stored as glycerol stocks at −80 °C and activated on appropriate media before experimental use. A full list of organisms, their sources, and culture conditions is provided in Supplementary Table S1. Optical density measurements and related inoculation procedures are detailed in Supplementary Table S2.

### 2.2. Preparation of Bacterial Inocula and OD600 to CFU Correlations

To ensure accurate bacterial inoculation counts, overnight cultures of all bacterial strains were prepared using Casamino acid–Peptone–Glucose [20] liquid broth supplemented with 6% yeast extract (CPGY) and incubated at 30 °C with shaking at 200 rpm. A 1 mL aliquot from each culture was transferred to a 1.5 mL Eppendorf tube and centrifuged at 15,000 rpm for 30 s to pellet the bacterial cells. The supernatant was removed and replaced with 1 mL of 1× Phosphate-buffered saline (PBS; Sigma-Aldrich, St. Louis, MO, USA). The tubes were vortexed to resuspend the cells and wash away the nutritive media. This washing process was repeated once more. One hundred microliters from each Eppendorf tube culture was then transferred to a sterile 96-well plate, and optical density at 600 nm (OD_600_) was measured using a BioTek Epoch 2 microplate reader (Agilent Technologies, Winooski, VT, USA). Serial dilutions of these Eppendorf tube cultures were performed, and 10 μL aliquots of these dilutions were plated on CPGY agar plates to allow for calculating colony-forming units per milliliter.

### 2.3. Fabrication of Master for Casting Microfluidic Device

Masters used to repeatedly cast microfluidic devices were fabricated using standard photolithography techniques established by Duffy et al. [21]. Briefly, microfluidic channel designs were made using Computer-Aided Design (CAD) software (Adobe Illustrator version 29.8.1) and printed onto a piece of transparency paper. The transparency paper containing the designs acts as a photomask for creating positive reliefs of SU-8 (Kayaku, Westborough, MA, USA) on silicon wafers. In this study, two layers of positive reliefs were created sequentially on the same silicon wafer using the photomasks marked as “first layer” and “second layer” (Figure S1), respectively. The thickness of the two layers was determined by the speed of spin-coating SU-8 on silicon wafer and validated with a digital dial gauge. The thicknesses of the first and second layer were 50 µm and 250 µm, corresponding to the height of the microfluidic channel and the height of the input and output ports, respectively.

### 2.4. Preparation of Microfluidic Device

Microfluidic devices were fabricated using a SYLGARD 184 Silicone Elastomer Base and Curing Agent Kit (Dow, Midland, MI, USA) following the protocols established by Duffy et al. [21]. First, a total of 11 g SYLGARD mixture, in a 10:1 ratio of base to curing agent, was thoroughly mixed and degassed under a vacuum-sealed container for 40 min or until all air bubbles were removed. The SYLGARD mixture was then poured onto the silicon wafer master. Two pieces of transparency paper were placed on top of and beneath the master to prevent the SYLGARD mixture from contacting the hotplates or other surfaces. A tongue depressor was used to spread the SYLGARD mixture on the master and remove any air bubbles generated during the previous steps. To cure the SYLGARD mixture and ensure uniform thickness, a 13,608 g (30 lb) weight was placed on the upper transparency paper, and the entire assembly was heated on a hotplate at 80 °C for 4 h.

**Figure 1:**
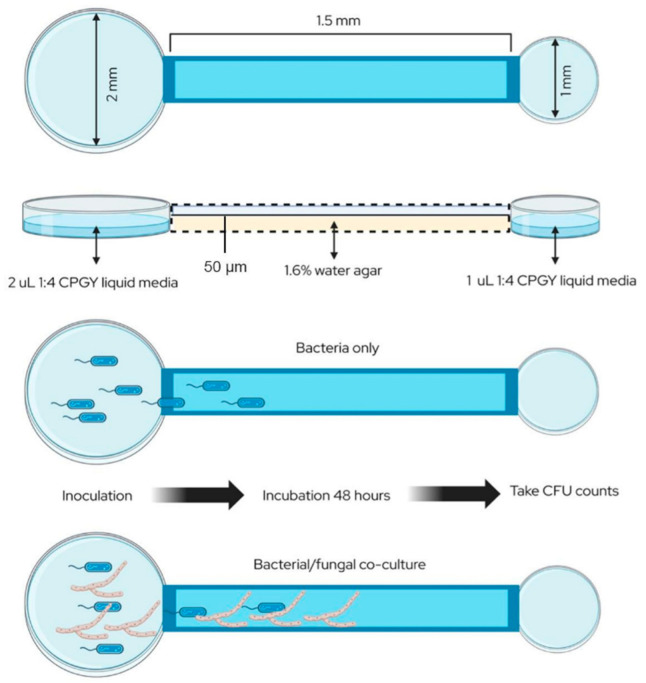
Depiction of microfluidic device. A strip of four microfluidic devices is bonded to a 35 mm glass-bottom Petri dish. The microfluidic channel consists of an input port joined by a thin channel filled with 1.6% water agar. The input port is inoculated with a bacterial monoculture or a bacterial–fungal coculture in diluted 1:4 CPGY media. Fluorinert FC-40 oil is then added to the Petri dish, submerging the channels to prevent drying.

Once cured, the solidified SYLGARD mixture was removed from the wafer and cut into strips of four microfluidic channels using a razor blade. Each microfluidic channel comprises an input port, where microbes are introduced, and an output port, connected by a microfluidic channel through which bacteria traverse (Figure 1). To bond the microfluidic devices in preparation for inoculation, 35 mm glass-bottom Petri dishes (Cellvis, D35-20-1.5-N) were used. The microfluidic devices were cleaned of any debris by applying scotch tape to the bottom side of the device. This was the side that made contact with the glass surface of the Petri dish. Once the devices were completely cleaned, they were placed, along with their corresponding Petri dish, channel side up, in a plasma etcher machine (Plasma Etch PE-50; Carson City, NV, USA) for 2 min. Upon completion, the devices were carefully removed from the plasma etcher and placed bottom side down on the glass portion of the Petri dish. The devices were gently pressed further onto the glass using a toothpick and allowed to continue bonding to the glass surface for an hour. To ensure sterile conditions, the plates were then UV-treated in a biosafety cabinet for 30 min.

### 2.5. Bacterial/Fungal Dispersal Assays

All bacterial–fungal cocultures were conducted using *A. flavus* strains. Fungal strains were first streaked from glycerol stocks onto glucose minimal media agar plates and incubated at 30 °C for two days. Following incubation, spores were harvested by suspending them in a 0.01% Tween 80 solution. The resulting suspension was passed through a 40 µm cell strainer into a sterile 50 mL conical tube. Spore concentrations were then quantified using Cellometer™ X2 fluorescent cell viability counter (Revvity, Lawrence, MA, USA) and adjusted to 1.25 × 10^4^ spores/mL, yielding a working solution with approximately 13 spores per microliter.

For each bacterial species, CPGY plates were inoculated from glycerol stocks, and isolated colonies were subsequently transferred into 14 mL tubes containing 3 mL of CPGY liquid medium. From these cultures, 1 mL was transferred to a 1.5 mL Eppendorf tube and centrifuged at 15,000 rpm for 30 s to pellet the cells. The supernatant was removed by pipetting and replaced with 1 mL of sterile 1× PBS. This washing step was repeated once to eliminate residual media. OD_600_ readings were then taken, and the bacterial suspensions were diluted to a concentration of 2.5 × 10^3^ cells/μL for inoculation. For bacterial strains without established OD_600_-to-CFU correlations, standard curves (Supplementary Table S2) were created by sampling overnight cultures at regular time intervals. At each interval, OD_600_ readings were taken while simultaneously performing serial dilutions for plating on CPGY agar. After incubation, colonies were counted to determine the CFU/mL corresponding to each OD_600_ value, allowing a reliable estimate of bacterial concentrations for subsequent experiments.

### 2.6. Inoculation of the Microfluidic Device

To prepare the microfluidic device for inoculation, a 1.6% water agar medium was prepared and sterilized. While still molten, approximately 150 μL of the agar was drawn into a pipette tip and dispensed into each of the four microfluidic channel input ports within a glass-bottom Petri dish, ensuring that the channels were filled. The agar was allowed to solidify for about one minute. Any excess agar that entered the input or output ports was carefully removed using a sterile pipette tip. To the input port of each microfluidic device, 2 μL of a 1:4 water dilution of CPGY liquid medium was added, followed by 1 μL to the output port. The following procedures were then carried out under either monoculture or coculture conditions:

Bacterial–fungal coculture: A 2 μL aliquot of spore suspension, containing 30 *A. flavus* spores, was introduced into the input port. Four microfluidic channels within a single 35 mm glass-bottom Petri dish were then submerged in 750 μL of sterile FC-40 oil to prevent desiccation. An additional 2 μL of bacterial suspension (2.5 × 10^3^ cells/μL) was subsequently added to the input port through the oil layer. Devices were incubated at 30 °C for 48 h.

Bacterial monoculture: As a control, input ports were inoculated with 2 μL of the bacterial suspension (2.5 × 10^3^ cells/μL) using the same method as the coculture setup, but without adding fungal spores from the corresponding fungal–bacterial pairings.

### 2.7. Assessment of Bacterial Dispersal

After 48 h of incubation, bacterial presence at the output port was assessed by pipetting away the FC-40 oil, then carefully withdrawing 1 μL of fluid from the output and transferring it into an Eppendorf tube containing 97 μL of sterile water. To ensure recovery of any remaining bacteria, an additional 2 μL of sterile water was pipetted into the emptied output port, gently aspirated to dislodge residual cells, and combined with the initial suspension. From this resulting mixture, a serial dilution was prepared by transferring 10 μL into 90 μL of sterile water and mixing thoroughly. This step was repeated sequentially five times, producing dilutions ranging from 10^−1^ to 10^−5^.

To prevent fungal growth while establishing bacterial CFU counts in bacterial fungal cocultures, CPGY agar supplemented with cycloheximide (100 µg/mL) was prepared. Subsequently, 10 μL of the various coculture and monoculture dilutions were plated onto a CPGY (+cycloheximide) agar plate. These plates were incubated at 30 °C for 2 days. CFUs were counted to determine the bacterial load in the output port of the microfluidic device used.

### 2.8. Selective Isolation of Listeria monocytogenes and Staphylococcus aureus in Triculture Experiments

To enable quantification of *L. monocytogenes* and *S. aureus* in triculture experiments with *A. flavus*, selective plating strategies were employed. *Listeria* was isolated by plating serial dilutions on CPGY agar supplemented with streptomycin (100 µg/mL) and incubating at 30 °C for 24–48 h. Under these conditions, *S. aureus* did not grow. Conversely, *S. aureus* was selectively isolated by plating it on mannitol salt agar (MSA) according to the American Society for Microbiology protocol and incubating at 37 °C [22], where Listeria did not form colonies. To validate the selectivity of each condition, control plates were inoculated with glycerol stocks of the species targeted for exclusion. *S. aureus* did not grow on streptomycin-supplemented CPGY at 30 °C, and *Listeria* did not grow on MSA at 37 °C, confirming the reliability of these conditions for differential enumeration.

### 2.9. Quality Control of Agr QS Phenotypes in Staphylococcus aureus Mutants

*S. aureus* mutants lacking a functional Agr QS system are prone to spontaneous reversion, which can restore Agr-mediated activity. To minimize this possibility, all strains were assessed for hemolytic activity using blood agar plates. Briefly, frozen glycerol stocks were first streaked onto CPGY agar plates containing 5% (*w*/*v*) defibrinated sheep blood and incubated at 37 °C for 48 h. Hemolysis was evaluated visually to confirm that wild-type strains exhibited robust β-hemolysis, while *agr* mutants showed no hemolysis. All strains were then inoculated into CPGY liquid medium and cultured overnight at 37 °C. Before microfluidic inoculation, cells were pelleted and washed twice with sterile PBS. To confirm that Agr-mediated activity had not re-emerged during processing, 10 µL of each PBS-washed culture was plated again onto blood agar and incubated at 37 °C for 48 h to reassess hemolytic activity. Only cultures that retained their expected hemolytic phenotype were used in downstream experiments (Figure S2).

### 2.10. Imaging and Image Analysis

All imaging was performed on a Nikon Eclipse TI inverted microscope (OKO Labs, Burlingame, CA, USA) at 10 or 20× magnification with the Nikon Plan Fluor lens system, using RFP, GFP, and DAPI channels to view fluorescently tagged or stained samples. Image analysis was done using the Nikon NIS Elements AR software package (Version 5.30) and Fiji (Version 2.9.0/1.53t).

### 2.11. Statistical Analysis

Data distribution and variance were assessed using the Shapiro–Wilk test and F-test to determine the appropriate statistical approach. Depending on these results, comparisons were performed using unpaired *t*-tests, Welch’s *t*-tests, or Mann–Whitney U tests, and descriptive statistics (mean ± SD) were calculated for all groups. For experiments involving two categorical variables, two-way ANOVA was conducted to evaluate main and interaction effects on CFU counts, followed by Tukey’s HSD post hoc analysis to determine specific pairwise differences. Statistical analyses were performed using R (v2023.12.1+402) and GraphPad Prism (v10.4.1), and significance was reported using standard GraphPad conventions: ns (*p* > 0.05), * (*p* ≤ 0.05), ** (*p* ≤ 0.01), *** (*p* ≤ 0.001), and **** (*p* ≤ 0.0001).

### 2.12. Figure Preparation

Schematic diagrams, illustrations, and graphical elements used in this study’s figures were created using BioRender.com. Agreement number: SH295MKXY6.

## 3. Results

### 3.1. Aspergillus flavus Enhances Dispersal of Multiple Bacterial Species Through Microfluidic Channels

**Figure 2:**
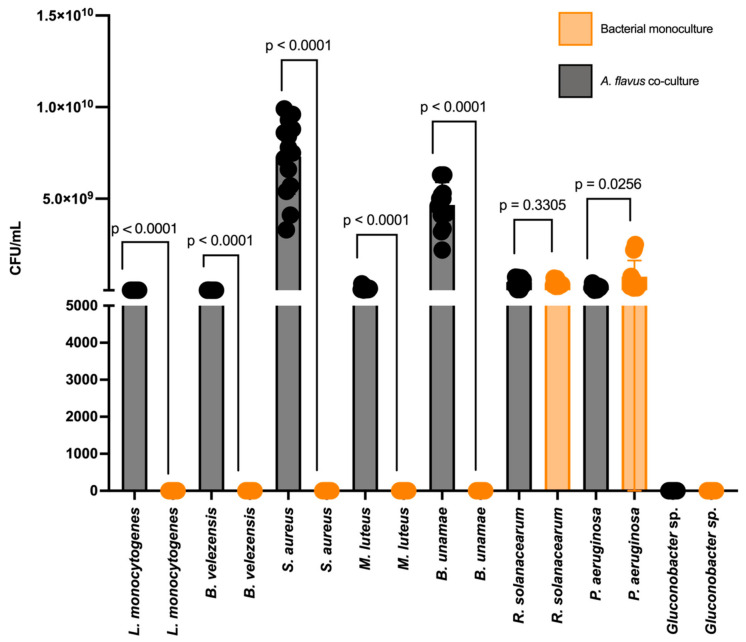
*Aspergillus flavus* enhances dispersal of multiple bacterial species through microfluidic channels. Eight bacterial species were cocultured with *A. flavus*. Of these, only *Gluconobacter* sp. failed to travel to the output port in either monoculture or coculture conditions. Five other bacterial species exhibited significantly enhanced dispersal when cocultured with *A. flavus*, including *L. monocytogenes*, *B. velezensis*, *S. aureus*, *M. luteus*, and *B. unamae. P. aeruginosa* showed a decrease in dispersal in coculture, and *R. solanacearum* exhibited similar dispersal rates in both monoculture and coculture conditions. All comparisons were analyzed using unpaired, two-tailed Mann–Whitney tests.

To gauge whether *A. flavus* influences bacterial dispersal through an agar substrate, eight bacterial species were cocultured with *A. flavus*. Their movement through a microfluidic channel was quantified by CFU counts at the device’s output port (Figure 1). These values were then compared to CFU counts from monocultures of the same bacterial species grown under identical conditions (Figure 2).

In monoculture, only *R. solanacearum* and *P. aeruginosa* reached the output port in appreciable numbers (Figure 2). *R. solanacearum* and *P. aeruginosa* both reached the output port in monoculture and coculture at fairly comparable levels, although *P. aeruginosa* was slightly limited in coculture. Five species—*L. monocytogenes*, *B. velezensis*, *S. aureus*, *M. luteus*, and *B. unamae*—only dispersed successfully in the presence of the fungus. *Gluconobacter* sp. failed to reach the output port under either condition. Overall, all four Gram-positive species tested (*L. monocytogenes*, *B. velezensis*, *S. aureus*, and *M. luteus*) were only able to reach the output port in coculture. Among Gram-negative species, three of the four tested—*B. unamae*, *P. aeruginosa*, and *R. solanacearum*—were able to traverse the channel, while *Gluconobacter* sp. did not.

### 3.2. Flagella Are Critical for Listeria monocytogenes but Not Pseudomonas aeruginosa or Ralstonia solanacearum Dispersal

**Figure 3:**
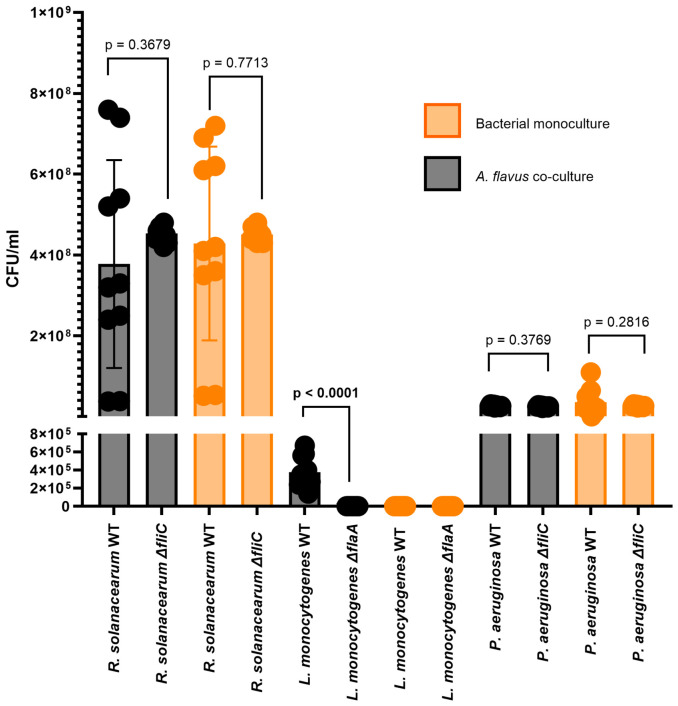
Flagellar dependency of *Listeria* dispersal in fungal coculture. *L. monocytogenes* wild-type (WT) but not a flagella mutant (*ΔflaA)* dispersed in coculture, while monocultures did not reach the output port. In contrast, *P. aeruginosa* and *R. solanacearum* showed no significant differences in dispersal between wild-type (WT) and flagella mutants (*ΔfliC*) under either monoculture or coculture conditions. All comparisons were analyzed using unpaired, two-tailed Mann–Whitney tests.

As several previous studies with bacterial species such as *B. subtilis* and *Salmonella enterica* have shown a requirement for flagella in bacterial dispersal [23,24], we examined this requirement in three different species. Wild-type and flagella mutants included a Gram-positive peritrichous species (*L. monocytogenes ΔflaA*), a Gram-negative monotrichous species (*P. aeruginosa ΔfliC*), and a Gram-negative peritrichous species (*R. solanacearum ΔfliC*). There was no significant difference in dispersal between wild-type and *ΔfliC* mutants under monoculture or coculture conditions for either *R. solanacearum* or *P. aeruginosa* (Figure 3). In contrast, *L. monocytogenes* showed a significant difference in dispersal between wild-type and *ΔflaA* strains in coculture, with only wild-type cells reaching the output port. As expected, neither *L. monocytogenes* wild-type nor *ΔflaA* strains reached the output port in monoculture; however, microscopy of GFP wild-type cells, but not *ΔflaA L. monocytogenes* monoculture cells, showed that they dispersed partway into the microfluidic channel before aggregating and forming a biofilm rim. Once this rim formed, bacterial cells could not advance further into the channel (Video S1). These findings suggest that flagella are critical for dispersal in *L. monocytogenes* but are not required in *P. aeruginosa* and *R. solanacearum* under the tested conditions (Figure 3).

### 3.3. Motility Barriers and Flagellar Function Define Temperature-Dependent Dispersal of Listeria monocytogenes

**Figure 4:**
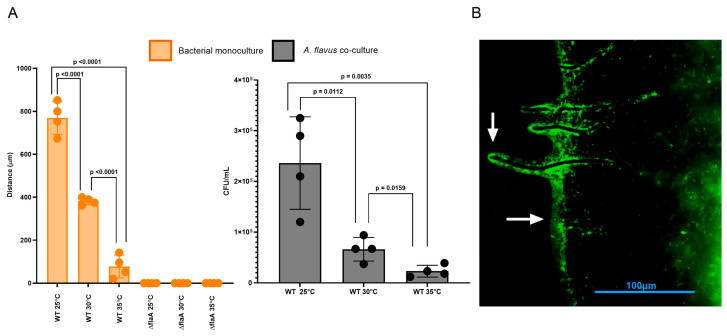
Temperature-dependent flagellar motility governs *Listeria monocytogenes* movement. (**A**) Dispersal capacity of *L. monocytogenes* was assessed by measuring the distance traveled through a microfluidic device in monoculture and coculture with *A. flavus* at 25 °C, 30 °C, and 35 °C. In monoculture, wild-type (WT) cells progressed partly down the microfluidic channels until forming biofilm barriers with significantly greater dispersal at decreasing temperatures. *∆flaA* flagellar mutants failed to disperse at any temperature. In coculture, WT *L. monocytogenes* cells reached the output port in higher numbers at decreasing temperatures. (**B**) *A. flavus* hyphae become enveloped by *Listeria* cells when they breach *Listeria* biofilm barriers. White arrows highlight the *Listeria* barriers and hyphae coated with *Listeria* cells (Video S2). All statistical analyses were done using unpaired *t*-tests with Welch’s correction.

It is known that *L. monocytogenes* motility and flagellar transcription are temperature-dependent and that *L. monocytogenes* cells demonstrate increased motility at lower temperatures [25]. We thus considered it possible that *L. monocytogenes* would disperse at a higher rate in lower temperatures. In comparing motility at 25 °C, 30 °C, and 35 °C, we found the bacterium dispersed further down the device both in monoculture and coculture with *A. flavus* (Video S1), while only reaching the output port in coculture (Figure 4). When *A. flavus* hyphae penetrated the biofilm rim formed by *L. monocytogenes*, the hyphae became surrounded by bacterial cells, which remained associated with the hyphae as they continued to grow and advance beyond the bacterial rim (Figure 4, Video S2).

### 3.4. Deletion of mogR Enables Independent Channel Traversal by Listeria monocytogenes

**Figure 5:**
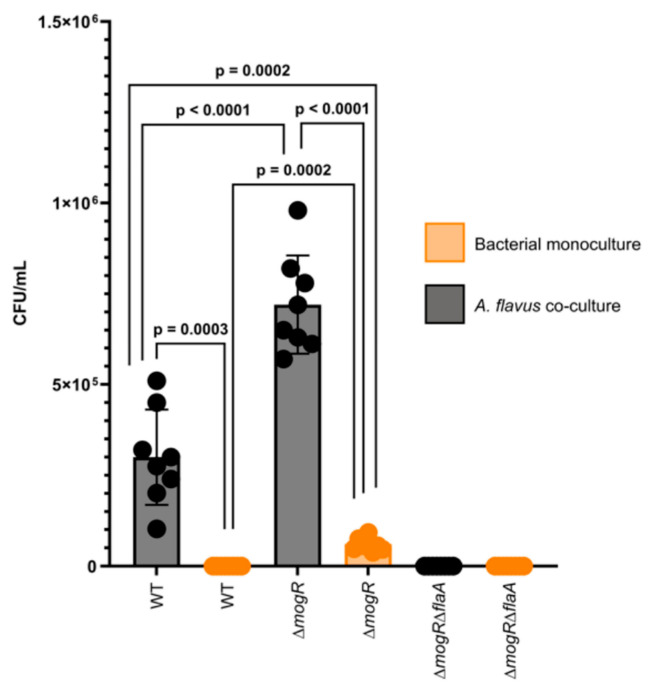
Deletion of *mogR* increases *Listeria monocytogenes* motility. CFU counts from the microfluidic channel output show that at high temperatures (35 °C) WT *L. monocytogenes* required coculture to reach the output port, whereas *∆mogR* mutants reached the output port in both monoculture and coculture. Both *∆mogR* monocultures and cocultures yielded significantly higher CFUs than WT monoculture and cocultures. Loss of flagella restricted all dispersal in a *∆mogR* background. Statistical analysis was done using unpaired *t*-tests with Welch’s correction.

In *L. monocytogenes*, the transcriptional repressor MogR binds to the promoter regions of flagellar genes, repressing their expression as the temperature nears human body temperature (37 °C) [19]. This regulation helps the pathogen evade the host immune response by limiting the production of flagellar-based pathogen-associated molecular patterns (PAMPs). We hypothesized that the deletion of ∆*mogR* may enhance *L. monocytogenes* dispersal in the microfluidic channel by permitting flagella production at higher temperatures. To test this, *∆mogR* and *∆mogR∆flaA* double mutants were examined in both monoculture and fungal coculture conditions within the microfluidic device at 35 °C. *∆mogR* mutants were able to traverse the entire microfluidic channel as a monoculture, albeit in low numbers—unlike the wild-type strain, which required fungal coculture for full channel dispersal (Figure 5). As expected, the *∆mogR∆flaA* double mutant remained stationary at the inoculation site, due to its inability to form flagella. When cocultured with *A. flavus*, the *∆mogR* mutant displayed higher CFU recovery at the output port compared to its wild-type parent (Figure 5). Taken together, these results implicate MogR as a potential fitness factor in microbiome ecologies.

### 3.5. Quorum-Sensing-Regulated Surfactant Activity May Mediate Staphylococcus aureus Dispersal

**Figure 6:**
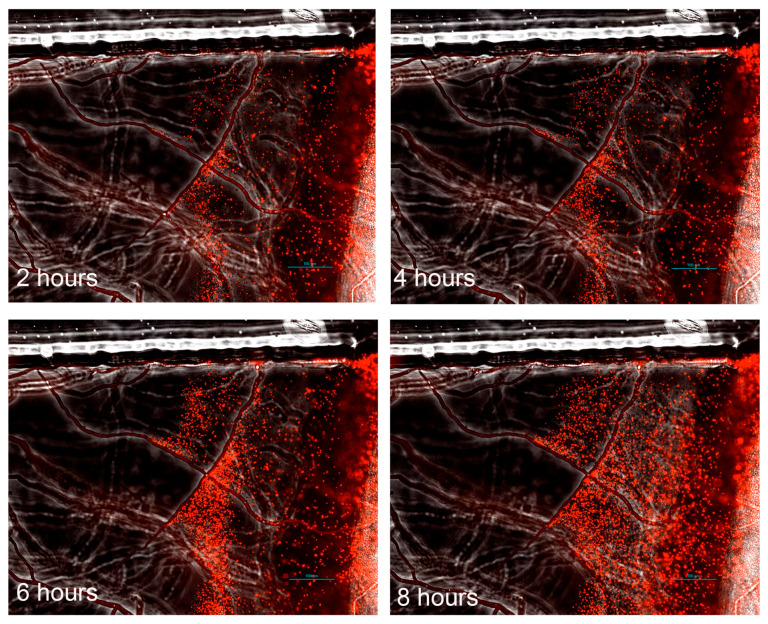
Hyphal branches create dispersal launch sites for *Staphylococcus aureus*. RFP-tagged *S. aureus* FPR3757LAC cells exhibit clumping behavior between the hyphae of *A. flavus*. The fungal hyphae form a crisscrossed, fork-like structure that serves as a focal point for bacterial aggregation. After approximately 7 h of congregation at this site, we observed a rapid dispersal of the bacterial population over the course of 14 min (further shown in Video S3).

In our initial experiments, non-flagellar bacterial species *M. luteus* and *S. aureus* successfully traversed to the output port in coculture but not in monoculture (Figure 2). To further investigate how a non-motile species disseminated within the microfluidic device, we used time-lapse microscopy imaging of RFP labeled *S. aureus* in coculture (Video S3 and Figure 6). In contrast to the movement of *L. monocytogenes* along individual hyphae of *A. flavus* (Video S4), *S. aureus* primarily accrued as clumps between crisscrossing or branching fungal hyphae (Figure 6). Remarkably, as the concentration of *S. aureus* cells increased, a rapid wave-like dispersal of *S. aureus* bacteria in the microfluidic device was repeatedly observed (Video S3).

**Figure 7:**
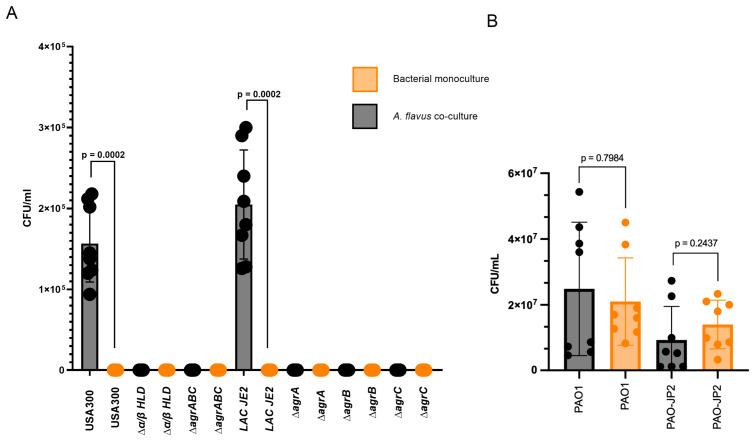
Quorum-sensing is essential for *Staphylococcus aureus* but not *Pseudomonas aeruginosa* dispersal. Two *S. aureus* strains (USA300 and LAC JE2) reach the output port only in coculture. Coculture dispersal was eliminated in all quorum-sensing mutants for both strains, including *Δagr*, *ΔagrA*, *ΔagrB*, *ΔagrC*, and Δ⍺/β HLD. (**A**) In contrast, both wild-type (PAO1) and a QS mutant (PAO-JP2) of *P. aeruginosa* were able to disperse in both monoculture and coculture (**B**). All comparisons were analyzed using unpaired, two-tailed Mann–Whitney tests.

Previous studies have shown that phenol-soluble modulins (PSMs) are small proteins that facilitate biofilm formation and the spread of *S. aureus* in wet environments [26]. These PSMs are known to induce wave-like detachment of *S. aureus* biofilms [27]. Given this, we hypothesized that PSMs may contribute to the wave-like dispersal of *S. aureus* within fungal networks. PSM synthesis contributing to this phenomenon is encoded by three genes, *psmα, psmβ,* and *hld,* which are subject to quorum regulation through the ag*rBDCA* operon [28]. Here, we examined dispersal using two different strains of *S. aureus*: one (USA300) lacking either a functional quorum system (*∆agr*ABC) or the ability to synthesize PSMs (*∆⍺/β HLD*) and the other (LAC JE2) lacking individual *agr* genes (*∆agrA*, *∆agrB*, *∆agrC*). As expected, all QS and PSM mutants failed to leave the initial inoculation port and did not disperse on fungal hyphae or in monoculture (Figure 7A). These findings indicate that a functional QS system and the downstream production of PSMs are necessary for the dispersal phenotype we observed in *S. aureus* across fungal networks. The importance of QS may be species-specific, as a similar analysis of a wild-type vs. a QS mutant of *P. aeruginosa* did not show any statistical difference in bacterial dispersal (Figure 7B).

### 3.6. Interbacterial Competition Along the Fungal Networks

**Figure 8:**
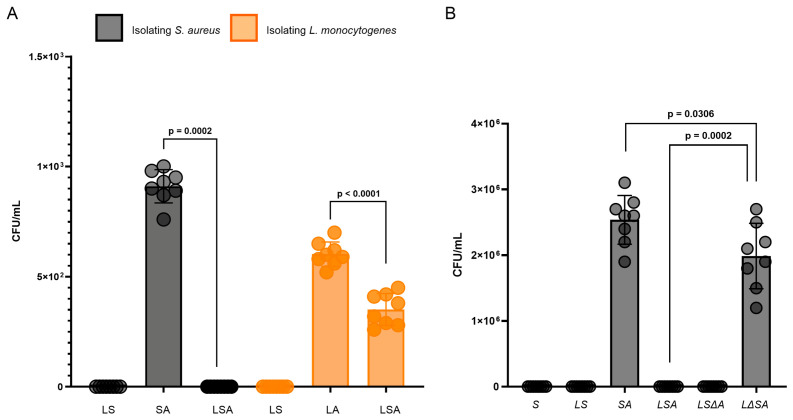
*Listeria monocytogenes* inhibition of *Staphylococcus aureus* dispersal requires an intact quorum-sensing system. (**A**) *S. aureus* is not detected at the output port when competing with WT *L. monocytogenes*. (**B**). *S. aureus* is detected at the output port when competing with the quorum-sensing *Δagr L. monocytogenes* mutant. LS = *L. monocytogenes* + *S. aureus*; SA = *S. aureus* + *A. flavus*; LSA = *L. monocytogenes* + *S. aureus* + *A. flavus*; LA = *L. monocytogenes* + *A. flavus*; S = *S. aureus*; LS∆A = *L. monocytogenes* + *Δagr S. aureus* + *A. flavus*; L∆SA = *ΔagrD L. monocytogenes* + *S. aureus* + *A. flavus*. Gray shows *S. aureus* counts and orange shows *L. monocytogenes* counts. All comparisons were analyzed using unpaired, two-tailed Mann–Whitney tests.

Multi-microbial communities have been shown to exhibit emergent behaviors that differ significantly from those observed in monoculture vs. cocultures, as interactions among species can alter growth dynamics, metabolic activity, and competitive outcomes [29]. For instance, cocultures of *L. monocytogenes* and *Bacillus cereus* demonstrate both cooperative and competitive interactions as assessed by *L. monocytogenes* biofilm formation [30]. We thus asked if the presence of two bacteria could change dispersal dynamics on fungal hyphae. As seen in Figure 8, when both *L. monocytogenes* and *S. aureus* were cultured with *A. flavus,* only *Listeria* was recovered in the output port. Microscopy indicated that RFP *S. aureus* cells were stuck in the input channel.

We thought it possible that this inhibition could be mediated by QS interference, as many studies have demonstrated QS cross-species antagonism [31,32]. Pertinent to our work, a recent study demonstrated that *L. monocytogenes* antagonizes *S. aureus* Agr signaling through its own Agr quorum-sensing system [33]. Considering that the Agr system is required for *S. aureus* dispersal (Figure 7), we asked if a functional *Listeria* Agr system was required for *S. aureus* suppression. As shown in Figure 8B, deletion of the Agr system (*∆agrD*) in *L. monocytogenes* restored the ability of *S. aureus* to disperse along fungal hyphae. These results indicate that a functional quorum-sensing system in *L. monocytogenes* is necessary for the competitive suppression of *S. aureus* dispersal in this triculture system.

## 4. Discussion

In this study, the dispersal patterns of various bacterial species along fungal networks were explored. The results demonstrated notable variability in the dispersal capabilities of different species that were not correlated with Gram-positive and Gram-negative status but rather appeared species-specific. We focused our studies on two Gram-positive pathogens that have been associated with fungi. *Listeria* is found in similar dairy environments as *A. flavus* [34] and *S. aureus* in medical settings with *A. fumigatus* and *Candida* spp. [35]. We found a requirement for flagella activity in *L. monocytogenes* and uncovered a possible relationship between QS-dependent movement on fungal hyphae, which warrants further investigation.

The striking difference in the ability of *L. monocytogenes, P. aeruginosa*, and *R. solanacearum* to reach the output port in monoculture vs. coculture led to an examination of the importance of flagella for dispersal in these three bacteria. The loss of flagella significantly impaired dispersal in *L. monocytogenes*, suggesting that flagellar motility is critical for its movement in this system (Figure 3). This follows the requirement of flagella for several other bacteria [13,14]. In contrast, the dispersal of *P. aeruginosa* and *R. solanacearum* flagellar mutants was largely unaffected, implicating alternative motility strategies. One such strategy could be type IV pili-mediated twitching motility, well established in both species [36,37].

The temperature-dependent movement of *L. monocytogenes* in monoculture and coculture highlighted the interplay between environmental conditions and bacterial motility. Temperature-dependent flagella movement is a known trait of *Listeria* [25]. Following this, we found that dropped temperatures might promote movement in both monoculture and coculture. In all temperatures tested in monoculture, *L. monocytogenes* formed dense biofilm rims of bacterial cells within the microfluidic channel, which occurred further into the channel at lower temperatures (Figure 4). When cocultured with *A. flavus, L. monocytogenes* also showed higher dispersal rates at lower temperatures. This dependency on flagella was supported by the increased bacterial dispersal in *ΔmogR* mutants, which lack repression of flagellar genes (Figure 5).

In *A. flavus*–*L. monocytogenes* interactions, fungal hyphae became coated with bacteria as they penetrated the biofilm rims (Figure 4). Here, the hyphae appeared to offer a physical scaffold, as has been observed with other bacterial/fungal pairings [38]. Strikingly, *L. monocytogenes* cells were observed gliding rapidly along fungal hyphae (Video S4), an active behavior absent in *ΔflaA* and *ΔmogRΔflaA* double mutants, suggesting a flagella-dependent mechanism for this kinetic movement.

Movement along fungal hyphae by the non-motile species *S. aureus* presented an alternative mechanism. Time-lapse microscopy showed that *S. aureus* cells became trapped between hyphal branches (Figure 6). As the bacterial concentration reached a threshold, a wave-like dispersal pattern emerged reminiscent of density-dependent quorum mechanisms involving the production of PSMs (Video S3), which are known to facilitate bacterial movement for this bacterium [26]. We found that *S. aureus* mutants lacking a QS apparatus or the ability to synthesize PSMs failed to disperse under any conditions. Although PSMs are amphipathic peptides known to impact virulence in disease and structure biofilm formation, our work supports the hypothesis that the basal role of these peptides is to support surface colonization of environmental niches, including fungal hyphae [39].

While monoculture studies reveal baseline motility traits, exploring bacterial cocultures on fungal hyphae highlights how inter-species interactions shape bacterial dispersal along fungal networks, reflecting conditions closer to natural microbial communities. Thus, we explored the potential of *L. monocytogenes* and *S. aureus* to impact the movement of each other along *A. flavus* hyphae. After finding that *S. aureus* was non-motile in the presence of *L. monocytogenes* (Figure 8), we speculated that QS systems could be involved based on previous research showing that *L. monocytogenes* produces an autoinducing peptide that inhibits the Agr QS system—and hence PSM production—in *S. aureus* [33]. Indeed, when the triculture included an Agr mutant of *Listeria, S. aureus* was able to again disperse on hyphae (Figure 8). These results illustrate that chemical antagonism of competing QS systems may impact bacterial dispersal on fungal networks in an environmental niche.

Although the microfluidic system enabled controlled visualization of bacterial dispersal along fungal networks, it cannot fully reveal the physical and chemical complexity of natural host-associated environments. The agar-filled channels and confined space may influence nutrient availability, hydration, and microbial behavior differently than open environmental systems. In addition, while devices were submerged in oil, we cannot completely exclude the possibility that agar matrix disruption by fungal hyphae or microscale pressure differentials contributed to bacterial dispersal within the device. However, the observation that several bacterial mutants failed to disperse in either monoculture or coculture argues against passive bulk flow as the primary movement mechanism. Furthermore, the molecular basis of several observed behaviors, including the rapid gliding-like movement of L. monocytogenes along hyphae and the precise role of fungal-derived factors in promoting or restricting dispersal, remains unresolved. Future studies combining high-resolution imaging and transcriptomic or genetic analyses could help define the physical and molecular mechanisms behind bacterial movement along fungal networks.

## Data Availability

The original contributions presented in this study are included in the article/Supplementary Material. Further inquiries can be directed to the corresponding author.

## Supplementary Materials

The following supporting information can be downloaded at: https://doi.org/10.5281/zenodo.20500678 (accessed on 1 January 2026).
